# Metagenomic Analysis Revealed Differences in Composition and Function Between Liquid-Associated and Solid-Associated Microorganisms of Sheep Rumen

**DOI:** 10.3389/fmicb.2022.851567

**Published:** 2022-05-27

**Authors:** Manchun Su, Ziyun Hao, Huibin Shi, Taotao Li, Huihui Wang, Qiao Li, Yong Zhang, Youji Ma

**Affiliations:** ^1^College of Animal Science and Technology, Gansu Agricultural University, Lanzhou, China; ^2^Gansu Key Laboratory of Animal Generational Physiology and Reproductive Regulation, Lanzhou, China; ^3^School of Agriculture and Forestry Technology, Longnan Teachers College, Longnan, China

**Keywords:** metagenomics, rumen microorganism, CAZymes, taxonomic diversity, functional diversity

## Abstract

The rumen microbiota plays a key role in the utilization of plant materials by ruminants, yet little is known about the key taxa and their genetic functions of the rumen sub-environment involved in the ruminal degradation process. Understanding the differences in the composition and function of ruminal microbiota in the liquid-associated (LA) and solid-associated (SA) systems is needed to further study and regulate rumen function and health. In this study, rumen contents of nine sheep were collected to separate LA and SA systems with elution and centrifugal precipitation. Metagenome sequencing was used to investigate the differences in microbial composition and genetic functions of LA and SA systems, with special emphasis on their degradational potential toward carbohydrates. Results showed that the dominant species composition was similar between the two systems, but SA microorganisms had a higher relative abundance than LA microorganisms in all taxa. The concentration of fiber-degrading bacteria, such as *Ruminococcus, Treponema*, and *Fibrobacter*, was higher and *Prevotella* was lower in the SA vs. LA system. Additionally, SA microorganisms dominated in cellulose degradation, while LA microorganisms were more important in starch utilization based on the Kyoto Encyclopedia of Genes and Genomes (KEGG) Orthology (KO)'s functional categories and Carbohydrate-Active Enzymes (CAZymes). In general, SA microorganisms are more abundant and important in metabolic functions than LA, such as carbohydrate and amino acid metabolisms. In summary, the key differential biomarkers between LA and SA systems were *Prevotella, Ruminococcus, Treponema*, and *Fibrobacter*. Ruminal microbes degraded carbohydrates synergistically with SA, thus, more focusing on cellulose and hemicellulose, while LA is more important to starch.

## Introduction

Ruminants are distinguished from other animals with their unique digestive organ—the rumen, which contains a complex and dynamic ecosystem with numerous microorganisms including bacteria, anaerobic fungi, protozoa, methanoarchaea, and bacteriophages. Ruminal microbes provide many essential nutrients to their host including microbial protein (MCP), volatile fatty acids (VFA), and vitamins (Elghandour et al., 2020).

Earlier studies on rumen microbes were mainly conducted using microscopic observation and microbial culture technology. Based on those research, rumen microbes are classified into three major groups: (1) liquid-associated (LA) microbes that suspend in rumen fluid, (2) solid-associated (SA) microbes that are associated with the feed particles, and (3) epithelium-associated (EA) microbes that colonize on the rumen epithelium (Wallace et al., 1979; McAllister et al., 1994). The SA microorganisms can be further divided into loosely associated microflora, which can be easily removed with a gentle wash, and tightly associated microflora, which cannot be removed. Larue et al. (2005) reported that SA microbiota is drastically different in composition and structure from LA microbiota, and their diversities can be influenced by the dietary composition. For instance, Sadet et al. (2007) used genetic fingerprinting and polymerase chain reaction-denaturing gradient gel electrophoresis (PCR-DGGE) techniques to study the dietary effects on rumen microbes and found the significantly distinct composition of EA, LA, and SA microbes in lambs fed on forages, but not in those fed on high concentrate diets. Studies using 16S rRNA gene clone library analyses also revealed the spatial distribution of rumen microbes. Several studies based on this technique have shown that rumen microbes are spatially differentiated (Kong et al., 2010; Pitta et al., 2010; De Menezes et al., 2011; Gharechahi et al., 2015; Mao et al., 2015). However, some studies argued that the differences between LA and SA are small and mainly exist in relative abundance (Petri et al., 2013; De Mulder et al., 2016; Ji et al., 2017).

Previous research mainly focused on rumen fermentation and microbial composition, but most of them failed to provide information on the metabolic functions of rumen microorganisms. Differences in rumen microbial composition indicate there might be different metabolic functions between microbes. Some scholars investigated functional differences in the rumen microbial ecosystem using Phylogenetic Investigation of Communities by Reconstruction of Unobserved States (Mao et al., 2015). Hinsu et al. (2020) and Pandit et al. (2018) have studied functional profiles in cattle and buffalo using metagenomic and metatranscriptomic approaches (Pandit et al., 2018; Hinsu et al., 2020). However, studies on functional differences between rumen LA and SA microorganisms are still missing in sheep. In addition, some reports in the field of biofuel research have shown that rumen SA microorganisms have a very important relationship with cellulose degradation (Brulc et al., 2009; Hess et al., 2011; Gharechahi et al., 2020, 2021). It is still unclear how different rumen LA and SA microorganisms are involved in carbohydrate degradation potential, and how the SA microorganisms contribute to CAZymes.

The microorganism of low abundance in the rumen may be easily ignored due to technical limitations of microbial culture and 16S rRNA gene sequencing. Metagenomics, on the other hand, not only enables the description of ruminal microorganisms at a greater resolution but also facilitates studying the linkage between nutrition and metabolism of the rumen microbiome at the genomic level (Lima et al., 2015; Pitta et al., 2016). In this study, we sequenced rumen microbes from 9 lambs and generated 202 gigabases of metagenomic sequential data to study the composition of rumen LA and SA microbes. In addition, we analyzed the functional differences in degrading carbohydrates between LA and SA ruminal microbes. To the best of our knowledge, this is the first study analyzing the differences in the composition and function of sheep rumen's LA and SA microorganisms using metagenomic techniques. We speculate that the microorganisms in the rumen sub-environment vary greatly, with microbes in rumen fluid being more biased toward soluble nutrients, while attached microbes are more capable of degrading cellulose. This study provides novel insights into the research of gastrointestinal microorganisms and acts as a guide for rumen sampling.

## Materials and Methods

### Ethics Approval

All experimental procedures involving animals were approved by the Animal Care Committee of Gansu Agricultural University (GSAU-AEW-2020-0057), in compliance with the animal care and experimental procedure guidelines established by the Ministry of Science and Technology of the People's Republic of China (Approval No. 2006-398).

### Animals and Experimental Design

Nine weaned Hu-sheep male lambs (aged 90 ± 5 days; 19.27 ± 0.45 kg body weight) from three pens were selected. The dietary ingredients and chemical composition of the basal diet are presented in Table 1. Lambs were housed in a barn with good ventilation and had *ad libitum* access to clean water. To avoid sorting and to maintain the desired forage to concentrate, a total mixed ration (TMR) was offered at 08:00 and 17:00 daily throughout the 60-day experimental period following 14 days adaptation period. Feed amounts were adjusted weekly to allow about 10% residues during the experimental period. TMR diet was sampled weekly and combined homogeneously at the end of the trial and analyzed for dry matter (DM), ash, crude fat, and crude protein according to the AOAC procedures (Cunniff and AOAC International, 1997). Acid detergent fiber (ADF) and neutral detergent fiber (NDF) were analyzed according to the procedures of Van Soest et al. (1991).

**Table 1:** Ingredients and nutrient composition of the basal diet [dry matter (DM) basis].

**Item**	**Diet**
**Ingredients (% of DM)**	
Ground corn	35.4
Wheat bran	10.5
Soybean meal	10.8
Rapeseed meal	4.3
Cottonseed meal	4.4
Oat hay	14.0
Alfalfa hay	13.3
Corn silage	4.1
Limestone	1.2
Salt	1.0
Vitamin-mineral mix^a^	1.0
**Nutrition composition^b^**	
DM, %	82.65
Crude protein, %DM	16.17
Crude fat, %DM	2.74
NDF, %DM	29.6
ADF, %DM	17.09
Ca, %DM	0.67
P, %DM	0.41

*DM, dry matter; NDF, neutral detergent fiber; ADF, acid detergent fiber*.

a *The premix provides the following nutrients per kg of diets: Fe 430 mg, Zn 950 mg, Cu 650 mg, I45 mg, Mn 600 mg, Se 30 mg, Co 20 mg, VA 120,000 IU, VE 8000 IU, VD 40,000 IU, and 2300 IU of VK*.

b *Measured values*.

### Sample Collection and Processing

All lambs were slaughtered in the early morning of day 61 at a local slaughterhouse (Qingyang, Gansu, China) at 12 h after receiving their previous afternoon feed. Rumen contents from each sheep were filtered through a four-layer sterilized cheesecloth. Three tubes of 50-ml rumen fluid and ~150 g of residues were kept in liquid nitrogen and later stored at −80°C before extraction of microbial DNA.

Rumen LA microorganism and SA microorganism were extracted by referencing the method from previous publications (Larue et al., 2005; Gharechahi et al., 2020). Briefly, rumen fluid was filtered with 4 layers of sterilized cheesecloth, and 50 ml of filtrate was centrifuged at 10,000 g for 20 min at 4°C. The supernatant was discarded, and the precipitation was suspended in 10-ml phosphate-buffered saline as rumen LA microorganism samples and stored at −80°C. Rumen residue of 10 g was thawed at 4°C and transferred to a 50-ml centrifuge bottle, and 30 ml of phosphate-buffered saline was added to resuspend the plant particles. The mixture was gently shaken for 60 s, then, centrifuged at 500 g for 15 min at room temperature to sediment the plant particles. The supernatant was carefully removed and transferred to another 50 ml of freshly sterilized bottles and the microbial cells (loosely adherent fraction, LAF) were harvested by centrifugation at 10,000 *g* for 20 min at 4°C. The remaining residue was resuspended in 50 ml of an anaerobically prepared diluent containing 0.15% (v/v) Tween-80, then, gently shaken for 60 s and placed on ice for 2.5 h to elute the tightly adherent bacteria. After removing the plant particles by centrifugation at 500 g for 15 min at room temperature, the microbial cells (tightly adherent fraction, TDF) were recovered by centrifugation at 10,000 *g* for 20 min at 4°C. The LAF and TDF microbes were re-suspended in a 10-ml phosphate-buffered saline (PBS) as rumen SA microorganism samples and stored at −80°C prior to analysis.

### Microbial DNA Extraction and Metagenome Shotgun Sequencing

Metagenomic DNA was extracted from the microbial samples using the Bacterial DNA Kits, following the instruction of the supplier (Omega, Shanghai, China). The DNA concentration was determined with Nanodrop 1000 (Thermo Fisher Scientific, Wilmington, DE, United States), and DNA integrity was evaluated on a 1% agarose gel. Metagenomic DNA libraries were constructed using the TruSeq DNA Sample Prep kit (Illumina, San Diego, CA, United States) and were sequenced *via* paired-end chemistry (PE150) on the Illumina Hiseq X Ten platform (Illumina, San Diego, CA, United States) at Biomarker Technologies (Beijing, China).

### Metagenome Shotgun Sequencing Analysis

#### Genome Assembly

Metagenome sequencing of total DNA from 18 rumen samples generated ~202 gigabases (GB) of raw sequences data. There were 34 GB of data, which likely originated from the host and were excluded from the datasets. Raw reads were filtered using Trimmomatic with a quality cutoff of 20 and reads shorter than 30 bp were discarded. The reads considered from the host were removed using DeconSeq v0.4.3 (Schmieder and Edwards, 2011) and bowtie2, with the National Center for Biotechnology Information (NCBI) Tibetan sheep genome sequences as references. The remaining high-quality reads of all samples were assembled by multiple megahits using Megahit V1.1.2(Li et al., 2015), which makes use of succinct de Bruijn graphs. Contigs of 300 bp and over were selected as the final assembly result. Results were evaluated by Quast (Zhu et al., 2010) and then contigs were used for further gene prediction and annotation. The statistical summary of multiple megahits is shown in Supplementary Table 1.

#### Non-redundant (NR) Gene Set Construction

Based on the assembly results, MetaGeneMark (http://exon.gatech.edu/meta_gmhmmp.cgi, Version 3.26) using default parameters predicted the presence of open reading frames. The summary statistics for gene prediction are shown in Supplementary Table 2. Predicted genes from all samples were gathered together to form a large gene set. MMseqs2 (https://github.com/soedinglab/mmseqs2, Version 11-e1a1c) was used to construct the NR gene set by setting 95% identity and 90% coverage of the gene with the longer sequences in the clustering.

#### Gene Abundance Calculation

Bowtie2 v2.3.4 was used to map clean reads of each sample to the NR gene set (Langmead and Salzberg, 2012). MarkDuplicates in the Picard toolkits version 2.0.11 was used to remove the PCR duplicates in the reads, and HTSeq v0.9.1 was then employed to calculate gene counts (Anders et al., 2015). Transcripts per kilobase of exon model per million mapped reads (TPM) of the gene, calculated as [(gene count/gene length) × 10^6^/sum (gene count/gene length)], were used to normalize gene abundance. The abundance of genes was compared using the R program DESeq2 package (Love et al., 2014). The differences were considered significant when the false discovery rate (FDR) was <0.05.

### Gene Taxonomy and Function Annotation

For taxonomic analysis, DIAMOND (diamond v0.9.29.130, e-value ≤ 1e^−5^) was used to blast the unigenes against the bacteria, fungi, archaea, and virus sequences in the Non-Redundant Protein Sequence Database (NR database) of the NCBI (https://www.ncbi.nlm.nih.gov/). The most similar sequence in the NR database was found, and the annotation information corresponding to the sequence is the annotation information corresponding to the sequenced genome gene.

#### KEGG Pathway Enrichment Analysis

The KEGG Orthology (KO) terms of predicted genes were obtained using the KEGG Automatic Annotation Server (KAAS) (Moriya et al., 2007). Subsequently, KO terms were mapped to KEGG pathways *via* the KEGG mapper provided by the KEGG website. The KEGG annotations were conducted using Diamond against the KEGG database (Version: 2018-07-30) with the e-value cutoff of 1e^−5^.

#### CAZymes Annotation and Taxonomic Profiling

The CAZymesannotation was conducted using hmmscan (Version 3.1b2) against the CAZy database Version 6.0 with the e-value cutoff of 1e^−5^. Putative plant cell wall polysaccharide-degrading enzymes belonging to different CAZy families were identified and classified according to sequence-based annotation. CAZymes encoding contigs were analyzed manually for different classes of CAZymes: Glycoside Hydrolases (GHs), Glycosyl Transferases (GTs), Carbohydrate Esterases (CEs), Carbohydrate-Binding Modules (CBMs), Auxiliary Activities (AAs), and Polysaccharide Lyases (PLs). Subsequently, CAZy results were analyzed to determine the proportions of different CAZymes present in the rumen metagenome data.

### Data Accession Number

The Illumina sequencing raw data for our samples have been deposited in the NCBI. The raw data are available under BioProject, accession number: PRJNA795697 (Metagenome).

### Statistical Analysis

Taxonomic and functional data analyses were performed using the online platform of BMKCloud (www.biocloud.net). Community richness and diversity, such as Chao1, ACE, Simpson, and Shannon indices, which were used to illustrate significant differences between two systematic samples, were assessed by the program MOTHUR v.1.35.0. Principal coordinate analysis (PCoA) for beta diversity was constructed and visualized using the R package (R ade4 package, version 2.15.3). The significance between groups in the PCoA plot was tested with permutational multivariate analysis of variance (PERMANOVA). Differential abundance of phylum, family, genus, CAZymes, and KO modules were analyzed by Wilcoxon test using stats R package in R software (version 3.3.1). Statistical significance was declared at 0.01 ≤ *P* < 0.05 “*” and *P* < 0.01 “**.” Linear discriminant analysis (LDA) effect size (LEfSe) analysis was used to determine the biomarkers explaining the differences between the LA and SA microbes. LDA score cut-off was set at 3.

## Results

### Analysis of Microbial Structure in Ruminal LA and SA Microorganisms

#### Sequencing Data

We assembled 5,371,141 contigs and identified 487 archaeal species from 102 genera and 14,990 bacterial species from 2,226 genera and 999 unclassified species.

#### Taxonomy of the Ruminal LA and SA Microorganism

**Figure 1:**
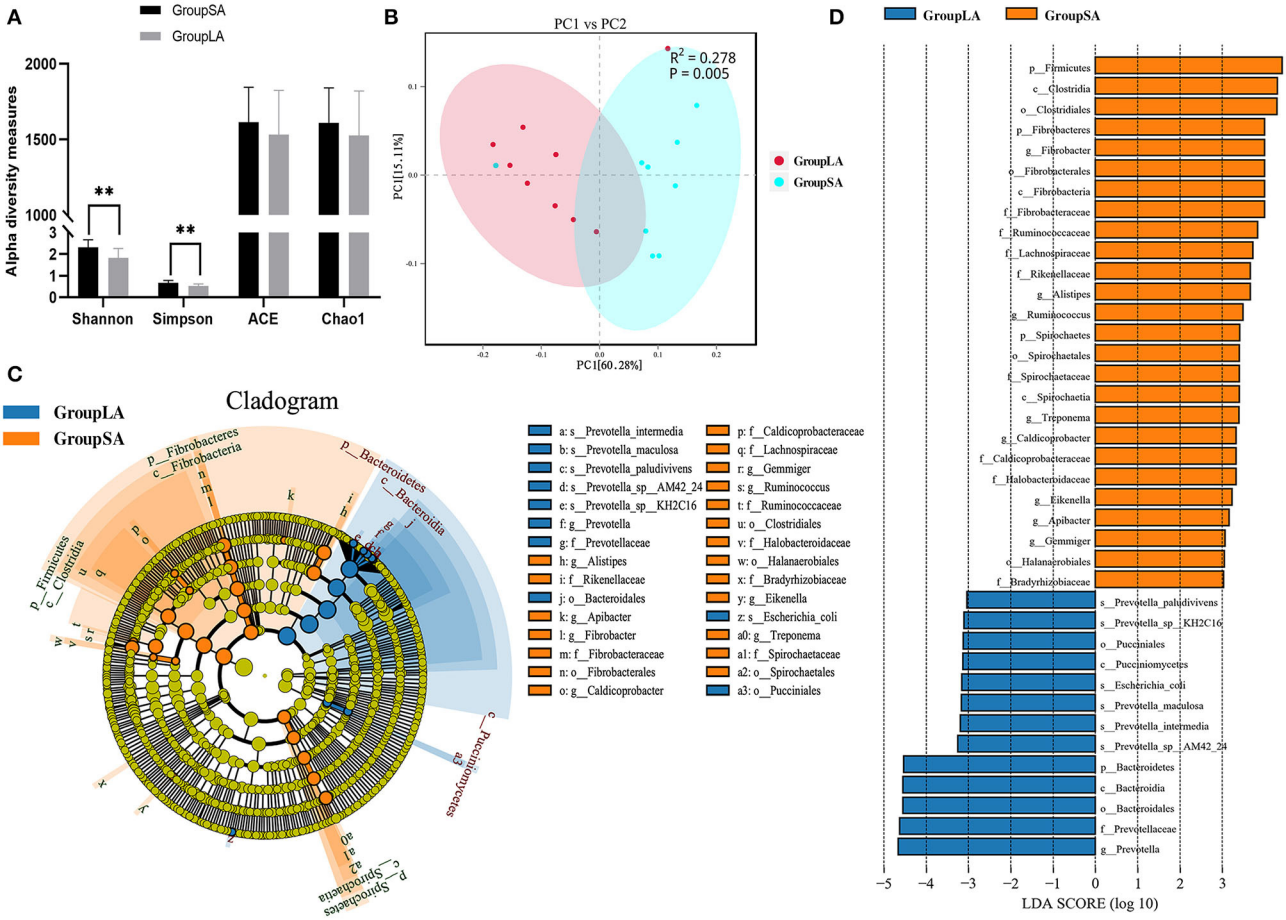
**(A)** Ruminal bacterial alpha diversity of liquid-associated (LA) and solid-associated (SA) microorganisms. **(B)** Beta diversity: principal coordinate analysis (PCoA) of bacterial community structure based on Bray–Curtis distances for LA and SA microorganisms. **(C)** Linear discrimination analysis (LDA) effect size (LEfSe) analysis comparing LA and SA groups. **(D)** Histogram of LDA scores computed for microorganisms abundant differently between LA and SA microorganisms. LDA scores (log10) > 3 were listed (*n* = 9 per group). ^*^P < 0.05, ^**^P < 0.01.

The ACE and Chao1 were indices of species richness, while Shannon and Simpson were indices of species richness and evenness. In this study, ruminal microbial alpha diversity was measured by Chao1, ACE, Simpson, and Shannon indices for both SA and LA microorganisms. Shannon index of the SA group was higher than the LA group (*P* < 0.01), similar to the Simpson index (Figure 1A). However, there was no difference in ACE and Chao1 indices between SA and LA microbes. These results indicated that microbial evenness and diversity of SA microbes were higher than those of LA microbes. Beta diversities of microbial communities between SA and LA were calculated and visualized by PCoA using the Bray–Curtis distance, and PERMANOVA was used to test significances. Results showed that microbial communities were distinct between LA and SA systems (Figure 1B). In general, SA microbes have more species and higher relative gene abundance at all taxonomic levels than LA microbes. At the species level, SA annotated 7,897.7 species, which was 12.79% higher than LA (Supplementary Table 3). It is noteworthy that one sample was anomalous in both SA and LA systems (Supplementary Figures 1–3), which might be caused by the individual differences of sheep or potential contamination of this sample. This phenomenon was also found in the subsequent functional analysis but did not affect the overall analysis.

The student's *t-*test was used to compare microbial communities between rumen SA and LA systems. The top 10 species at 6 taxonomic levels are shown in Table 2. For the annotated species, at the phyla level, Bacteroidetes (54–63% of relative abundances), Firmicutes (14–21%), Proteobacteria (1.9–2.7%), Spirochetes (0.3–3.2%), and Fibrobacteres (0.6–1.6%) were the dominant phyla. Compared with the LA group, the SA group had a higher relative abundance of Firmicutes, Spirochaetes, and Fibrobacteres, and a lower relative abundance of Bacteroidetes (*P* < 0.05). Class level taxonomy showed that the most abundant classes were Bacteroidia (53–62% of relative abundances) and Clostridia (8–14%). The relative abundance of Clostridia, Spirochaetia, Fibrobacteria, and Flavobacteriia was higher, and Bacteroidia was lower for the SA group vs. LA group at class level (*P* <0.05). On the order level, Bacteroidales (52.8–62% of relative abundances), Clostridiales (8.3–13.,9%), and Spirochaetales (3.1%) were the most abundant orders. Higher (*P* < 0.05) relative abundance of Clostridiales, Spirochaetales, Fibrobacterales, Acidaminococcales, Flavobacteriales, and lower Bacteroidales were observed in the SA group compared with the LA group. Family level taxonomy showed that the most abundant classified families were *Prevotellaceae* (39.1–49.6% of relative abundances), *Bacteroidaceae* (4.8–5.0%), *Lachnospiraceae* (2.8–5.0%), *Ruminococcaceae* (2.2–4.4%), and *Spirochaetaceae* (0.3–3.1%), with the remaining families representing <3% on average of the relative abundances. Compared with the LA group at the family level, the SA group had a higher relative abundance of *Lachnospiraceae, Ruminococcaceae, Spirochaetaceae, Rikenellaceae*, and *Fibrobacteraceae*, and a lower relative abundance of *Prevotellaceae* (*P* < 0.05). On the genus level, *Prevotella* (36.6–47.5% of relative abundances), *Bacteroides* (4.7–4.9%), *Ruminococcus* (1.5–3.1%), and *Treponema* (0.2–3.1%) were the most abundant genera, with the remaining genera accounting for < 2% on average of the total read abundances (Table 2). A decrease (*P* < 0.05) in *Prevotella* and an increase in *Ruminococcus, Treponema*, and *Fibrobacter* were observed in the SA group compared with the LA group (Table 2). At the species level, we observed a higher relative abundance of *Treponema_bryantii* and *bacterium_P3* in the SA group, and higher *Prevotella_sp._AGR2160* and *Prevotella bryantii* abundance in the LA group (Table 2).

**Table 2:** Top 10 microbial players at six taxonomic levels in rumen SA and LA systems.

**Taxonomy**	**LA**	**SA**	**SEM**	***P*** **^a^**
**Phylum**				
*Bacteroidetes*	62.99	54.10	2.58	*
*Firmicutes*	13.87	21.11	1.37	**
*Spirochaetes*	0.34	3.17	0.33	**
*Proteobacteria*	2.68	1.90	0.59	NS
*Fibrobacteres*	0.56	1.64	0.25	*
*Euryarchaeota*	0.22	0.26	0.06	NS
*Tenericutes*	0.18	0.22	0.05	NS
*Actinobacteria*	0.17	0.19	0.04	NS
*Chlamydiae*	0.09	0.11	0.02	NS
*Fusobacteria*	0.06	0.07	0.01	NS
**Class**				
*Bacteroidia*	62.19	53.00	2.62	*
*Clostridia*	8.35	14.04	1.09	**
*Negativicutes*	3.63	4.57	0.59	NS
*Spirochaetia*	0.33	3.14	0.33	**
*Fibrobacteria*	0.56	1.64	0.25	*
*Gammaproteobacteria*	2.18	1.47	0.55	NS
*Erysipelotrichia*	0.38	0.44	0.05	NS
*Bacilli*	0.40	0.43	0.03	NS
*Flavobacteriia*	0.29	0.34	0.01	*
*Methanobacteria*	0.20	0.23	0.06	NS
**Order**				
*Bacteroidales*	62.02	52.80	2.62	*
*Clostridiales*	8.28	13.93	1.08	**
*Spirochaetales*	0.33	3.14	0.33	**
*Selenomonadales*	2.14	1.77	0.44	NS
*Fibrobacterales*	0.56	1.64	0.25	*
*Veillonellales*	0.74	1.39	0.32	NS
*Acidaminococcales*	0.70	1.35	0.21	*
*Aeromonadales*	1.42	1.01	0.46	NS
*Erysipelotrichales*	0.38	0.44	0.05	NS
*Flavobacteriales*	0.29	0.34	0.01	*
**Family**				
*Prevotellaceae*	49.55	39.06	2.75	*
*Bacteroidaceae*	4.80	5.03	0.23	NS
*Lachnospiraceae*	2.82	5.00	0.46	**
*Ruminococcaceae*	2.15	4.37	0.49	**
*Spirochaetaceae*	0.32	3.13	0.33	**
*Clostridiaceae*	1.63	1.95	0.26	NS
*Rikenellaceae*	1.05	1.85	0.32	NS
*Selenomonadaceae*	2.12	1.74	0.44	NS
*Fibrobacteraceae*	0.56	1.64	0.25	*
*Veillonellaceae*	0.74	1.39	0.32	NS
**Genus**				
*Prevotella*	47.51	36.62	2.87	*
*Bacteroides*	4.72	4.93	0.23	NS
*Ruminococcus*	1.48	3.16	0.41	*
*Treponema*	0.23	3.06	0.31	**
*Alistipes*	1.04	1.84	0.32	NS
*Clostridium*	1.51	1.78	0.25	NS
*Fibrobacter*	0.56	1.64	0.25	*
*Selenomonas*	1.53	1.21	0.34	NS
*Dialister*	0.55	1.12	0.27	NS
*Succiniclasticum*	0.46	0.91	0.21	NS
**Species**				
*Prevotella_multisaccharivorax*	17.44	8.89	1.61	NS
*Treponema_bryantii*	0.20	4.21	0.27	**
*Prevotella_sp._AGR2160*	6.06	3.52	0.45	*
*bacterium_P3*	1.31	3.10	0.29	*
*Prevotella_sp._tf2-5*	1.67	2.52	0.49	NS
*Prevotella_ruminicola*	2.13	2.40	0.24	NS
*Prevotella_sp._ne3005*	1.62	2.00	0.27	NS
*Ruminococcus_sp._FC2018*	0.83	1.88	0.23	NS
*Prevotella_bryantii*	2.36	1.55	0.14	*

*LA, liquid-associated microorganism; SA, solid-associated microorganism;*

a NS, not significant (P > 0.05); *(0.01 < P ≤ 0.05); **(P ≤ 0.01).

To further evaluate the differences in microbial communities between SA and LA systems and screen out the specific biomarkers, a linear discriminant analysis (LDA) effect size (LEfSe) was performed. Coupling standard tests were used for statistical significance with additional analyses examining biological consistency and effect relevance. Results showed that starch and protein degrading bacteria *Prevotella spp*. and glucosidase active bacteria *Bacteroidia* were significantly enriched in the LA system, whereas plant fiber degrading bacteria *Fibrobacteres* and *Clostridia*, resistant starch-degrading bacteria *Firmicutes*, and hemicellulose degrading bacteria *Spirochaetes* were significantly associated with SA system (Figures 1C,D). The above results disclosed the differences in microbial composition between rumen LA and SA systems, microorganisms enriched in the SA system were closely related to crude fiber degradation, while those enriched in LA were more related to starch and protein digestion.

### Rumen Microbial Function Between LA and SA Microorganisms

**Figure 2:**
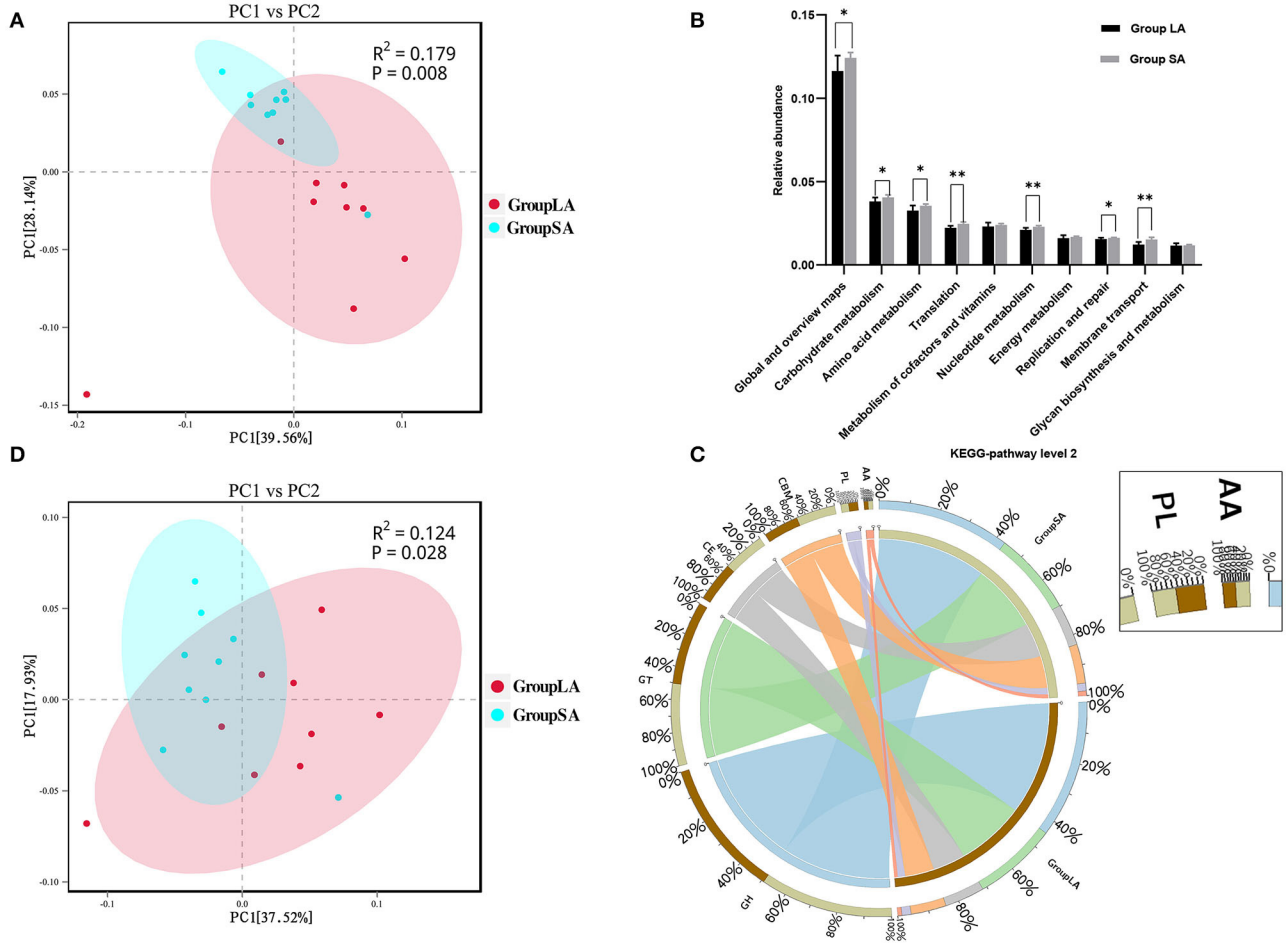
**(A)** Principal coordinates analysis (PCoA) of LA and SA systems in Kyoto Encyclopedia of Genes and Genomes (KEGG)-Orthology (KO) level. **(B)** Functional differences between LA and SA microorganisms at KEGG-pathway level 2. **(C)** Percentage of CAZymes in each group. **(D)** PCoA of LA and SA microorganisms at CAZy-family level (*n* = 9 per group). ^*^P < 0.05, ^**^P < 0.01.

At the gene level, a total of 2,631,315 non-redundant genesets were detected and were annotated to 6,279 KOs. Overall LA and SA fractions showed differences in KO levels as evidenced by the PCoA plot (Figure 2A). Compared to the LA group, relative abundances of 556 KOs were upregulated in the SA group, with only 5 KOs being downregulated (Supplementary Table 4). To further understand the functional differences between LA and SA microorganisms, students' *t*-test was used to evaluate their differences at KEGG-pathway level 2. Carbohydrate metabolism, amino acid metabolism, and translation represented the most abundant categories. Level 2 categories showed that most of the metabolism categories were abundant in SA. For the top 10 pathways, the relative abundance of 7 pathways in SA microbes was higher than in LA microbes (Figure 2B). These pathways are involved in carbohydrate, amino acid, and nucleotide metabolism, as well as translation function, replication and repair, and membrane transport.

The CAZy database defines six classes of enzymes involved in carbohydrate metabolism, including cellulases, hemicellulases, and amylases (Lombard et al., 2014). To specifically explore the microbial potential for dietary degradation in ruminal SA and LA systems metagenomes, we screened for CAZymes in the assembled contigs. CAZymes belonging to different classes (GHs, GTs, CEs, CBMs, PLs, and AAs) are shown in Figure 2C. Among these six classes of CAZymes families, glycoside hydrolases (GHs, 43.7%) were the most abundant family, and auxiliary activities (AAs,1.4%) were the least abundant family. GHs hydrolyze glycosidic bonds in complex carbohydrates and often assist in the degradation of cellulose, hemicellulose, and starch (Supplementary Figure 4). To evaluate the capacity of carbohydrate degradation of rumen LA and SA microbes, the PCoA method was used to their differences in CAZymes, and PERMANOVA was used to test the significance. The student's *t*-test was used to further compare the detailed differences between SA and LA in the CAZy class level. As shown in Figure 2D, microbial CAZymes were distinct between LA and SA systems (*P* < 0.05). Carbohydrate-binding modules (CBMs) and AAs' relative abundance in the SA group were higher than in the LA group (*P* < 0.05) (Table 3). The AAs' class in CAZy includes a number of enzymes acting in conjunction with GHs, GTs, CEs, and PLs classes, while CBMs are a class of proteins that assist carbohydrate enzymes to bind to their substrates.

**Table 3:** CAZymes contributing to GH, GT, CBM, CE, AA, and PL (all CAZymes were collectively considered 100%).

**Class**	**Description**	**LA**	**SA**	**SEM**	***P*** **-value**
AA	Auxiliary activities	1.30	1.54	0.002	**
CBM	Carbohydrate-binding modules	11.41	12.65	0.014	*
CE	Carbohydrate esterases	12.86	13.58	0.017	NS
PL	Polysaccharide lyases	2.96	2.53	0.014	NS
GH	Glycoside hydrolases	44.50	42.98	0.069	NS
GT	Glycosyl transferases	26.97	26.71	0.038	NS

LA, liquid-associated microorganism; SA, solid-associated microorganism; NS, not significant (P > 0.05); ^*^(0.01 < P ≤ 0.05); ^**^(P ≤ 0.01).

### Genes Related to the Carbohydrate Degradation Pathway

#### Phylogenetic Analysis of CAZymes

**Figure 3:**
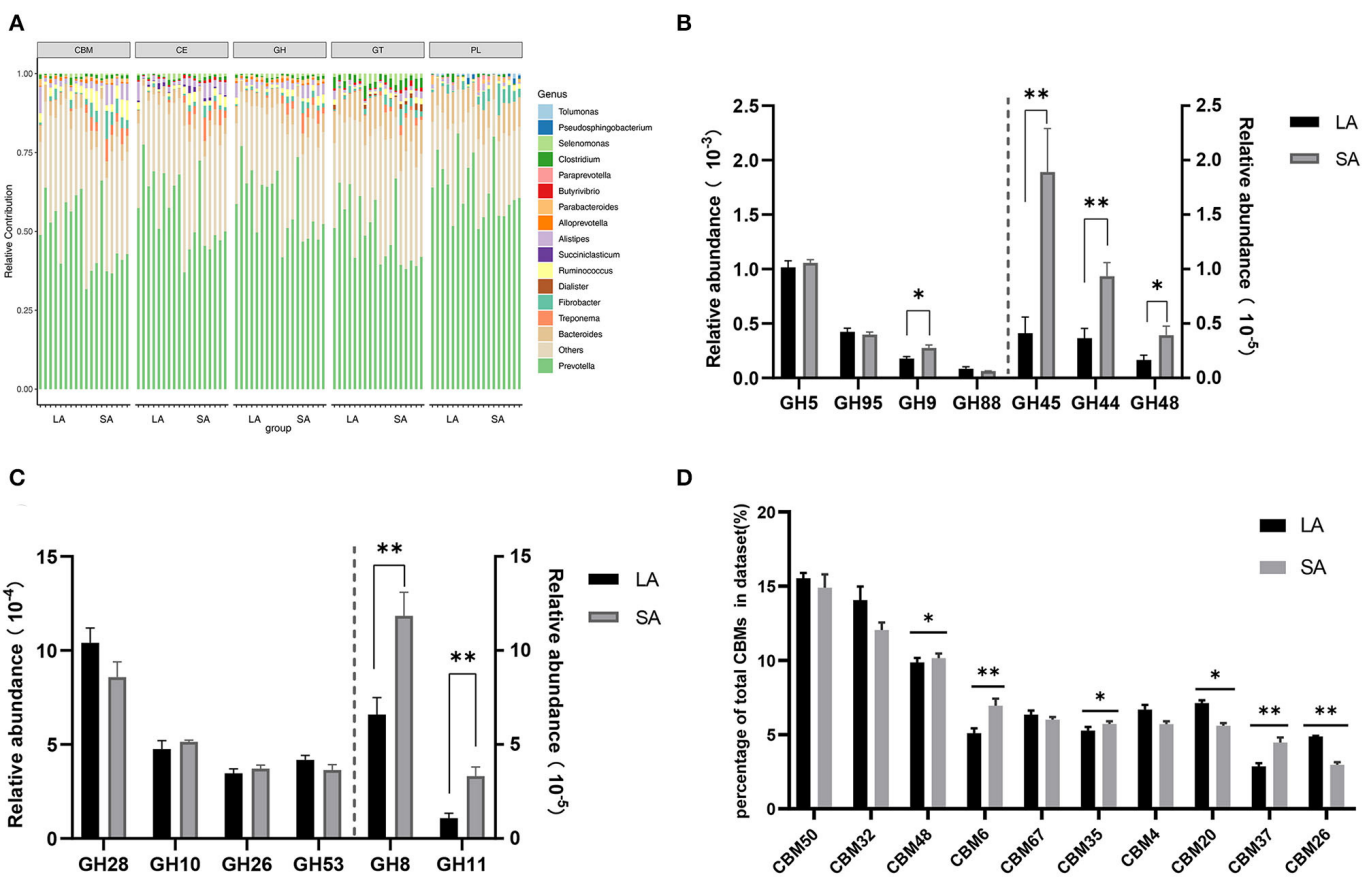
**(A)** Percent contributions of CAZymes from major microbial communities in LA and SA groups. GH stands for glycoside hydrolase, GT for glycosyltransferase, and PL for polysaccharide lyase. CE for carbohydrate esterases and CBM for carbohydrate-binding module. **(B)** Comparisons of gene abundance of GH family gene-coded cellulase in LA and SA groups. **(C)** Comparisons of gene abundance of GH family gene-coded hemicellulase in LA and SA groups. **(D)** Comparisons of gene abundance of top10 CBMs in LA and SA groups (*n* = 9 per group). ^*^P < 0.05, ^**^P < 0.01.

Carbohydrate degradation, especially cellulose, is one of the most important functions of rumen microorganisms. Therefore, it is necessary to further explore the differences in carbohydrate degradation potential between SA and LA systems. At the phyla level, phylogenetic analysis of CAZyme contigs showed that Bacteroidetes, Firmicutes, and Fibrobacteres primarily contributed to CAZyme-encoding gene fragments of the CBM, CE, GH, GT, and PL families in sheep rumen metagenome (Supplementary Figure 5). In these CAZymes families, Firmicutes, Fibrobacteres, Spirochaetes, and Proteobacteria were higher, while Bacteroidetes and Proteobacteria were lower in the SA group compared to the LA group (Supplementary Figure 5). Bacteroidetes, Firmicutes, Fibrobacteres, Spirochaetes, and Proteobacteria were found in all five CAZyme categories and were the primary contributors of CAZymes at the phyla level. At the genus level, phylogenetic analysis of CAZyme contigs showed that *Prevotella, Bacteroides*, and *Fibrobacter* primarily contributed to CAZyme-encoding gene fragments of CBM, CE, GH, GT, and PL families in rumen metagenome (Figure 3A). The number of CBM, CE, GH, and GT enzymes belonging to *Treponema* and *Fibrobacter* were significantly higher in SA than LA groups; the opposite was observed for *Prevotella* (Figure 3A). *Alistipes* was found in all five categories, with the highest abundance in CBM, CE, and GH families, followed by GT and PL families. The relative contribution of *Alistipes* to CBM, CE, and GH families was significantly higher in SA than in the LA group (Figure 3A). The number of enzymes from *Ruminococcus*, one of the most dominant bacteria of the CBM, CE, and GH families, was higher in SA than in the LA group. *Dialister, Succiniclasticum*, and *Clostridium* were also found in these five categories. *Dialister* was only distributed in GT, while *Succiniclasticum* was only distributed in CE, and *Clostridium* was mainly distributed in GT (Figure 3A). In summary, *Prevotella, Bacteroides, Fibrobacter, Ruminococcus*, and *Alistipes* were found in all five CAZyme categories and were the primary contributors of CAZymes in the genus level.

#### Comparison of CAZyme Families of Ruminal LA and SA Microorganism

To further explore the key carbohydrate biodegradation process of ruminal LA and SA microbes, we compared the GH families that are responsible for cellulose, hemicellulose, and oligosaccharide degradation. In our study, seven GH families (GH5, GH95, GH9, GH88, GH45, GH44, and GH48) were found to be mainly associated with cellulolytic functions (Figure 3B). The relative abundances of GH45, GH44, GH9, and GH48 were significantly higher in the SA group (*P* < 0.05), with 4.59, 2.55, 1.55, and 2.36 times higher compared to the LA group, respectively.

Six GH families (GH28, GH10, GH26, GH53, GH8, and GH11) were detected in our study to have important roles in hemicellulose degradation (Figure 3C). Compared with the LA group, we found that the abundance of GH8 and GH11 genes were 1.80 and 3.09 times higher in the SA group (*P* < 0.01).

Oligosaccharide-degrading enzymes are major parts of GH families, representing 44.86 and 45.69% of total GHs in LA and SA groups, respectively (Table 4). Compared with the LA group, we found that the abundance of GH39 and GH94 genes were significantly higher by 173.77% and 30.07% (*P* < 0.01), and GH13 and GH32 genes were significantly lower by 3.74 and 14.43% in the SA group (*P* < 0.05). The GHs that are responsible for oligosaccharide degradation (oligo-GHs) are mainly GH2, GH3, GH13, GH97, and GH43. The most abundant GH (7.50 and 8.16% of total GHs in LA and SA groups, respectively) was GH43 that encodes endoglucanase.

**Table 4:** Oligosaccharide-degrading and debranching enzymes were identified from LA and SA microorganisms.

**Enzyme and CAZy family**	**Major activity^a^**	**% of each group relative to total GHs identified in each dataset**			
		**LA**	**SA**	**SEM**	***P*** **-value**
**Oligosaccharide-degrading enzymes**					
GH1	β-glucosidases	0.22	0.28	0.001	NS
GH2	β-galactosidases	6.85	6.72	0.008	NS
GH3	β-glucosidases	5.99	6.35	0.006	NS
GH13	α-Amylase	7.15	6.88	0.005	*
GH18	Chitinase	0.87	0.92	0.002	NS
GH29	α-L-fucosidosis	1.21	1.41	0.003	NS
GH27	α-Galactosidase	0.59	0.66	0.001	NS
GH20	β-Hexosaminidase	1.08	1.14	0.004	NS
GH39	β-xylosidases	0.07	0.19	0.000	**
GH32	Invertase endo-inulinase	1.68	1.43	0.002	*
GH35	β-galactosidases	1.28	1.17	0.002	NS
GH31	α-Glucosidase	2.34	2.51	0.003	NS
GH38	α-mannosidases	0.17	0.17	0.001	NS
GH42	β-galactosidases	0.07	0.13	0.000	NS
GH43	arabino/xylosidases	7.50	8.16	0.011	NS
GH57	α-Amylase	0.94	1.00	0.001	NS
GH94	cellobiose phosphorylase	0.80	1.04	0.001	**
GH97	α-Glucosidase	3.16	2.92	0.005	NS
GH92	α-1,2-mannosidase	1.91	1.70	0.005	NS
GH130	β-1,4-Mannosylglucose phosphorylase	0.98	0.93	0.002	NS
**Debranching enzymes**					
GH23	Peptidoglycan lyase	2.17	2.47	0.003	NS
GH33	trans-Sialidase	0.60	0.70	0.001	NS
GH54	α-L-arabino furanosidases	0.01	0.04	0.000	*
GH51	α-L-arabino furanosidases	2.60	2.43	0.004	NS
GH67	α-glucuronidases	0.78	0.63	0.002	NS
GH77	4-α-Glucanotransferase	1.42	1.59	0.001	*
GH78	α-L-rhamnosidase	1.42	1.72	0.003	NS
GH84	N-Acetyl	0.15	0.15	0.001	NS
GH103	β-glucosaminidase transglycosylase	0.04	0.03	0.000	NS
GH127	α-Galactosidase	1.96	1.83	0.003	NS

LA, liquid-associated microorganism; SA, solid-associated microorganism; NS, not significant (P > 0.05); ^*^(0.01 < P ≤ 0.05); ^**^(P ≤ 0.01).

a *Major activity was mainly referenced by Wang et al. (2019)*.

Debranching enzymes were also identified belonging to GH23, GH33, GH51, GH54, GH67, GH78, GH78, GH84, GH103, and GH127 families. For this group, the most abundant GH (2.60 and 2.43% of total GHs in LA and SA groups, respectively) was GH51 that codes α-L-arabino furanosidase (Table 4). GH54 and GH77 were more abundant(*P* < 0.05) in the SA group than in the LA group, and the abundance of GH54 in the SA group was 8.61 times higher than in the LA group.

The CBM domain helps CAZyme binding to carbohydrate substrate, thereby facilitating the enzyme's activity (Boraston et al., 2004). We selected the top10 relative abundance CBMs for difference analysis (Figure 3D). Results showed that CBM50 was the most abundant family, which binds primarily to either chitin or peptidoglycan. Compared with the LA group, we found that the abundance of CBM48, CBM6, CBM35, and CBM37 genes were increased by 2.84, 36.40, 8.51, and 56.06% (*P* < 0.05), and CBM20 and CBM26 genes were decreased by 21.46 and 39.48%in SA group (*P* < 0.05). The CBM37, CBM35, and CBM6 exhibit rather broad binding specificity to xylan, chitin, microcrystalline, and phosphoric-acid swollen cellulose.

## Discussion

Ruminal microbiota contains a dense and complex mixture of bacteria, archaea, fungi, and protozoa that can convert plant biomass into VFAs and MCP providing to the host. One of the common methods to study rumen microorganisms is collecting rumen fluid. However, previous studies reported that microorganisms from different ruminal fractions including rumen epithelial tissue, rumen liquid, or solid fractions can be different (Kong et al., 2010; Mann et al., 2018). In the present study, we analyzed rumen microbial structure and metabolic functions of LA and SA microorganisms with metagenomics analyses and explored their degradational differences toward carbohydrates with sheep fed on a high-concentrate diet.

### The Microbial Composition Between LA and SA Microorganisms

Analysis of alpha and beta diversities of SA and LA microbes in this study showed that SA microbes had higher richness than LA microbes. A study on Holstein-Friesian dairy cows showed that Shannon-Wiener and Simpson diversity indices of a solid-adherent environment were significantly higher than that of a liquid environment (De Mulder et al., 2016), which is in line with our results. Although the dairy diet (forage-to-concentrate ratio of 70:30) was different from ours (forage-to-concentrate ratio of 30:70), similar results were observed, indicating that the compositional differences between rumen SA and LA microbes are independent of dietary concentration levels. Another study on Holstein cows reported that rumen bacterial diversity was mainly affected by diet and individual cows rather than rumen fractions (Ji et al., 2017), which may be related to the treatment of rumen samples. The rumen ecosystem is a dynamic system, in which suspending cellulolytic microbes in the liquid phase can adhere to feed particles, and metabolites produced by previous colonists attract secondary colonizers (Leng, 2014). After feed particles are fully degraded, all microorganisms attached to them will be released into rumen fluid. Therefore, each feed particle can be regarded as a small complex micro-habitat, gathering a variety of microorganisms. Feed particles stay in the rumen longer than rumen fluid, so the richness of microbial species in solid samples is more abundant than in the liquid. Although LA and SA environments are continuously interacted and mutually influenced, differences in microbial composition between them were reported in previous studies (Larue et al., 2005; Leng, 2011; De Mulder et al., 2016). However, most of the previous studies were carried out based on high forage diets and had limited studies on microbial genome sequencing.

With metagenomic techniques, we obtained different results from previous studies using microbial culture and 16S gene sequencing for species studies. A study based on 16S ribosomal RNA (rRNA) gene sequencing showed that solid-adherent and liquid environments exhibited no differences in taxonomic composition, but can be distinguished by the relative abundance of species (De Mulder et al., 2016). There was also a study on cattle that came to the opposite conclusion, demonstrating that liquid and solid fractions had distinct bacterial community patterns (Pitta et al., 2014). Our study showed that out of 166 microbial taxa found at the phylum level, LA and SA microbes shared 155 species, with 6 solely found in the LA system, and 5 only observed in the SA system (Supplementary Figure 6). At the microbial genus level, we identified 2,831 genera with LA microbes having 311 unique genera and SA having 198 unique genera (Supplementary Figure 7). These results suggest that LA and SA systems differ not only in the relative abundance of species but also in the taxonomic composition with distinctive species in both systems. At the phylum level, Bacteroidetes and Firmicutes were the two most abundant classifications in both systems, which agrees with the previous ruminant studies (Xu et al., 2021; Zhang et al., 2021). Bacteroidetes as one of the most abundant phyla in the rumen has a wide range of sugar degradation and protein hydrolysis potential, which is significant for the digestion and utilization of non-fibrous polysaccharides and proteins (Woodmansey, 2007; De Mulder et al., 2016). *Prevotella*, which is an important genus from the Bacteroidetes phylum with high capabilities of degrading protein and starch (Bandarupalli and St-Pierre, 2020; Shen et al., 2020), was found more abundant in the LA group in this study. *Ruminococcus* and *Fibrobacter* were more enriched in SA microbes, which can attribute to their needs of adhering to feed particles for dietary fiber degradation (McAllister et al., 1994). Interestingly, we found that Spirochaetes and *Treponema* were significantly enriched in SA samples, which is in line with a previous study on dairy cows (De Mulder et al., 2016). A possible explanation is that Treponema species, unable to utilize cellulose as a carbon source, may establish close synergistic relationships with certain cellulolytic bacteria to obtain soluble sugars released during fiber degradation (Kudo et al., 1987). We speculate that there might be more such synergistic bacteria attaching to feed particles alongside cellulolytic bacteria, using the secondary metabolites from cellulolytic bacteria as their nutrient source. A previous study using microbial culture and 16S RNA gene sequencing identified two *Treponema* isolates possessing low-cellulose degradation ability from the rumen of sheep (Nyonyo et al., 2014), suggesting that *Treponema* may also assist other bacteria utilizing cellulose substrates.

Diet is one of the important factors affecting rumen microbial diversity, due to microbial preferences for specific substrates. Previous studies have shown that high forage diet significantly increased the relative abundance of fibrobacterota (Pandit et al., 2018; Xu et al., 2021). Forages with different nutritional quality can also affect rumen microorganisms. Firmicutes, fibrobacterota, bacteroidota, ruminococcaceae, and spirochaetota prefer forages with high hemicellulose and low ADF, while verrucomicrobiota has a higher abundance of forages with high fiber content (Gharechahi et al., 2020, 2021). It is well known that diet alters ruminal microflora, but little is known about the effects of diet on LA and SA microbial communities. Ji et al. (2017) found that the segregation of SA and LA microbes was more pronounced in high fiber diets, but effects on specific species were not explored. Rumen SA microbial diversity of Holstein cows fed on high forage was higher than LA microbes, and SA microbes were richer in Lachnoaceae, Ruminpccaceae, Fibroacteraceae, and Spirochaetaceae, while LA was richer in Prevotellaceae and Succinibrionaceae (De Mulder et al., 2016). Similar results were observed on sheep fed on a high concentrate diet. We hypothesized that Rumen SA microorganisms are composed primarily of cellulose and lignin-degrading bacteria, while LA microbes consist primarily of members utilizing soluble carbohydrates. The division of these functional members is not affected by dietary factors, but their abundance may be affected. Unfortunately, we were not able to analyze the dietary effects on the abundance of these members due to the lack of a high-forage group. These results illustrate the importance of SA microorganisms and collecting only rumen fluid for ruminal degradation research may be biased. Additionally, sampling time also influences rumen microorganisms. Previous studies described the temporal shift from copiotrophy to oligotrophy in rumen microbial function with incubation length (Gharechahi et al., 2021). Eventually, the retained microorganisms are mainly those that can use refractory components.

### The Overall Function and Carbohydrate Degradation Potential Between LA and SA Microorganisms

The ability to perform functional analysis of microbial communities directly based on functional genes is one of the most important applications of the metagenomic technique. The present study indicates that SA microorganisms play more important roles in rumen metabolic function, supported by the research at the KEGG-pathway level 2. Earlier studies have shown that ruminal SA microbes are complex and diverse and may play an important role in rumen degradation (McAllister et al., 1994). In contrast, LA microbes may have limited contribution to metabolic activities and simply serve as a transit system of bacteria from SA biofilms prior to colonizing new feed particles (Leng, 2011). But a study on buffalo showed the opposite result, they indicated that most of the metabolism categories were abundant in liquid fraction (Hinsu et al., 2020). This may be because rumen samples were handled differently and because they collected rumen contents 3 h after feeding, whereas we collected them 12 h later. Its ability to degrade carbohydrates, especially plant cellulose, is the most important function of rumen microorganisms. We assessed the differences in carbohydrate degradation potential between LA and SA microbial systems. At the class level, the abundance of genes encoding CBMs and AAs was significantly higher in SA microbes than in their LA counterparts, suggesting that SA microbes may have a stronger ability to degrade carbohydrates, which is also consistent with the study on species taxonomy in this study. This is in line with previous studies on the function of microorganisms attached to forage in carbohydrates degradation (Hess et al., 2011; Stewart et al., 2018). Rumen metagenomic analysis showed that GH families were most abundant in SA and LA microbial systems of sheep, which was consistent with previous studies (Stewart et al., 2018; Lu et al., 2020). The GH family contains a large number of enzymes for polysaccharide degradation, such as cellulose, hemicellulose, and starch, and is one of the critical carbohydrase families for plant cell wall degradation (Hess et al., 2011; Lu et al., 2020). Phylogenetic analysis of CAZyme contigs showed that *Prevotella, Bacteroides, Treponema*, and *Ruminococcus* primarily contributed to CAZyme-encoding gene fragments of GH families in sheep rumen metagenome, which was consistent with previous studies with dairy goats (Shen et al., 2020) and Holstein cows (Wang et al., 2019). Among microorganisms encoding GHs gene fragments, *Prevotella* contributed more in LA systems, while *Treponema* and *Ruminococcus* contributed more in SA systems, which is also found in our species variability study. Microbial communities changed along with available substrates, similar to a previous study with cows fed on low-forage and high-forage diets (Wang et al., 2019), and consistent with the increase in *Prevotella* under low-fiber conditions.

Rumen microbiota is largely and functionally redundant with regard to carbohydrate degradation potential (Solden et al., 2018). Therefore, it may be more accurate to assess the carbohydrate degradation potential of SA and LA systems in terms of the abundance of specific substrate degrading enzymes. Based on linkages to specific substrate classes, Solden et al. (2018) assigned genomes into three trophic levels in the carbon food chain: (1) recalcitrant plant polymer degradation; (2) mixed polymer degradation and sugar fermentation; and (3) exclusive sugar fermentation. According to this theory, we performed a comparative analysis of three trophic levels in the GHs family: (1) cellulase; (2) hemicellulose; and (3) oligosaccharide-degrading enzymes and debranching enzymes. GH families, which are associated with three trophic levels in carbohydrate degradation functions, were identified by previous studies (Brulc et al., 2009; Wang et al., 2019; Shen et al., 2020) and CAZy Database (http://www.cazy.org/). In the present study, we found that GH5, GH95, and GH9 were the most abundant, which were also determined by previous studies (Wang et al., 2019; Shen et al., 2020). Notably, the abundance of genes encoding GH9, GH45, GH44, and GH48 were higher in SA microbes. GH9, GH44, and GH45 are well-known for endoglucanase activity (Maharjan et al., 2018; Wang et al., 2019), which suggests that rumen SA microbes may be more capable of degrading cellulose.

Hemicellulose is one of the most important components of plant cell walls, which consists of various polysaccharides, such as xylan, glucan, and mannans, among which xylan is the most abundant. The ability to degrade hemicellulose is important for ruminants to utilize the energies from forage. Our study identified six GHs families with hemicellulose degradation activity. Among these GHs families, GH28 was the most abundant, followed by GH10 and GH26. GH28 mainly encodes Polygalacturonase, which also plays an important role in pectin digestion (Zhao et al., 2014; Wang et al., 2019). GH10 is proposed to be closely related to xylanases, while GH26 is closely related to β-mannanase in addition to encoding Xylanases (Moraïs and Mizrahi, 2019; Wang et al., 2019). GH8 and GH11 families were annotated in the CAZy database as mainly associated with xylanases (http://www.cazy.org/), which indicates that SA microbes are more capable of degrading xylan. Oligosaccharide-degrading enzymes and debranching enzymes are the counterparts to the third trophic level of the carbon food chain proposed by Solden et al. (2018). Substrates of this trophic level consist mainly of single or oligomeric five- or six-carbon sugars. Organisms that do not express glycoside hydrolases to degrade plant polymers, express isomerase, or kinase to incorporate specific sugars into central metabolism (Solden et al., 2018). In the present study, up to 30 GH families encoding oligosaccharide-degrading and debranching enzymes were identified. The GH families encoding these two types of enzymes accounted for more than half of the entire GH family gene abundance, which was consistent with previous findings (Dishita et al., 2014; Wang et al., 2019). We found that abundances of GH13 and GH32 genes encoding α-Amylase and invertase were significantly higher in the LA system, whereas abundances of GH39, GH94, and GH77 genes encoding β-xylosidases, cellobiose phosphorylase, and 4-α-Glucanotransferase were higher in the SA system. It is interesting that the GH13 family, the most abundant GH family in this classification and the most important family encoding α-amylase, is more abundant in LA microbes to previous studies (Moraïs and Mizrahi, 2019; Shen et al., 2020). This suggests that microbes floating in rumen fluid can utilize starch more efficiently than those adhering to feed particles.

## Conclusion

In conclusion, we investigated the differences in species composition and metabolic functions between LA and SA microorganisms in the rumen of sheep fed on a high-concentrate diet using metagenomic techniques. Our results showed that there were differences in species classification and relative abundance between the two systems. *Ruminococcus, Treponema*, and *Fibrobacter* associated with fiber degradation were more enriched in the SA system, while *Prevotella* associated with protein and starch was more abundant in the LA system. The study of KO and CAZymes genes illustrated the differences between SA and LA systems in carbohydrate degradation. Genes related to cellulose degradation were more abundant in the SA system, while genes related to amylase were more abundant in the LA system. The present study enhances our understanding of the relationship between rumen microorganisms and their degradational functions. Future studies can focus on the change of feed-sensitive microbiota in different ruminal sub-habitat, which could help us regulate rumen health more precisely *via* feeding.

## Data Availability Statement

The datasets presented in this study can be found in online repositories. The names of the repository/repositories and accession number(s) can be found in the article/Supplementary Material.

## Ethics Statement

The animal study was reviewed and approved by Animal Care Committee of Gansu Agricultural University.

## Author Contributions

MS and YM contributed to the concept and the design. MS, ZH, HW, QL, HS, and YZ performed the research and analyzed the data. MS, TL, and YM wrote the manuscript. All authors contributed to manuscript revision, read, and approved the submitted version.

## Funding

This study was supported by the Education Science and Technology Innovation Project of Gansu Province (GSSYLXM-02) and the National Key R&D Program of China (2021YFD1100502). The funders had no participation in the study design, data collection and analysis, decision to publish, and preparation of the manuscript.

## Conflict of Interest

The authors declare that the research was conducted in the absence of any commercial or financial relationships that could be construed as a potential conflict of interest.

## Publisher's Note

All claims expressed in this article are solely those of the authors and do not necessarily represent those of their affiliated organizations, or those of the publisher, the editors and the reviewers. Any product that may be evaluated in this article, or claim that may be made by its manufacturer, is not guaranteed or endorsed by the publisher.

## Supplementary Material

Supplementary Table 1 Summary statistics of sample assembly results.

Supplementary Table 2 Summary statistics of sample genetic prediction results.

Supplementary Table 3 Comparison of taxonomy and relative abundance between LA and SA systems.

Supplementary Table 4 Effects of liquid-associated (LA) and solid-associated (SA) systems on relative abundances of KEGG Orthology (KO) terms in the ruminal microbiome (up/down means upregulation/downregulation in SA with comparison to LA).

Supplementary Figure 1 Principal coordinate analysis (PCoA) of bacterial community structure based on Bray–Curtis distances for LA and SA microorganisms (with individual labels).

Supplementary Figure 2 PCoA analysis of LA and SA systems at KO level (with individual label).

Supplementary Figure 3 PCoA analysis of LA and SA microorganism in CAZy-family level (with individual label).

Supplementary Figure 4 Diagram of carbohydrate enzyme distribution in all samples (*n* = 18).

Supplementary Figure 5 Percent contributions of CAZymes from the microorganisms at phylum level in LA and SA groups. GH stands for glycoside hydrolase, GT for glycosyltransferase, and PL for polysaccharide lyase. CE for carbohydrate esterases, CBM for carbohydrate-binding module.

Supplementary Figure 6 Venn diagram of the microbial composition of SA and LA systems at the phylum level.

Supplementary Figure 7 Venn diagram of the microbial composition of SA and LA systems at genus level.
