# Supplementary figures and images for: Metagenomic Analysis Revealed Differences in Composition and Function Between Liquid-Associated and Solid-Associated Microorganisms of Sheep Rumen
Source: Front Microbiol. 2022 May 27;13:851567. doi: 10.3389/fmicb.2022.851567 (PMC9197192; doi:10.3389/fmicb.2022.851567)

# PCoA - PC1 vs PC2

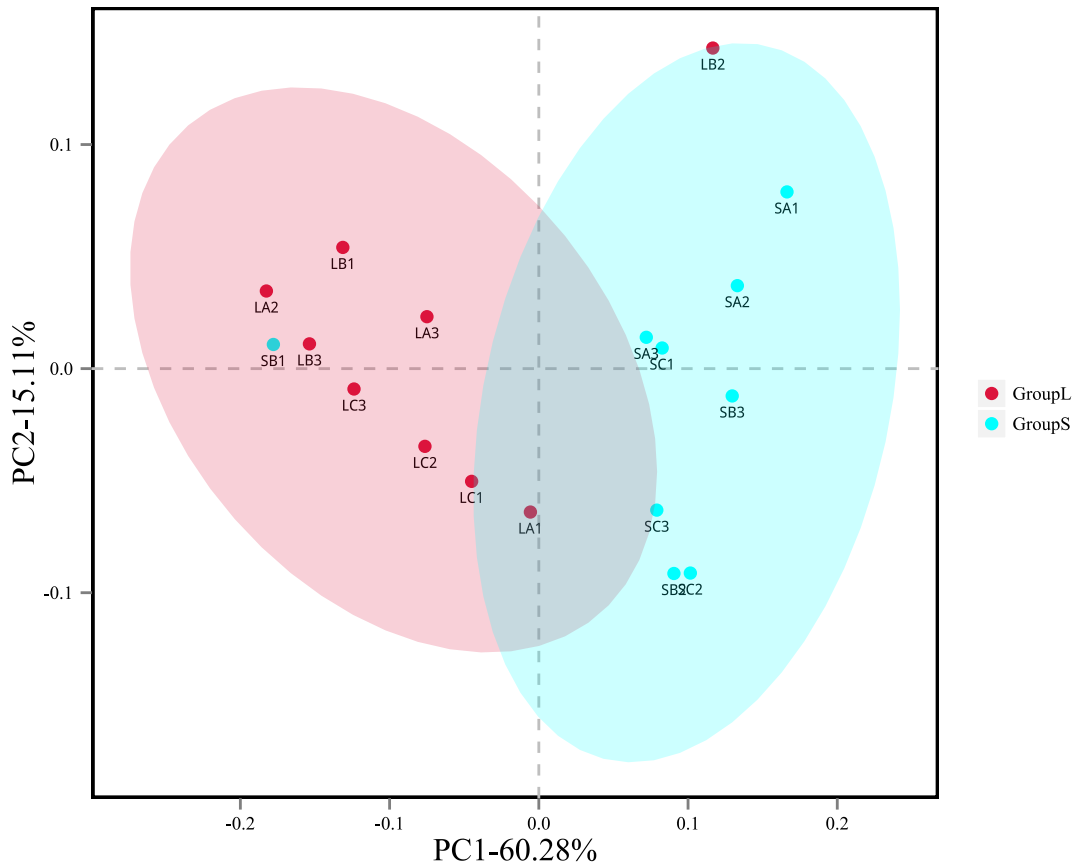

Supplement: Supplementary Figure 1 — Principal coordinate analysis (PCoA) of bacterial community structure based on Bray–Curtis distances for LA and SA microorganisms (with individual labels). [file Image_1.pdf]

PCoA - PC1 vs PC2

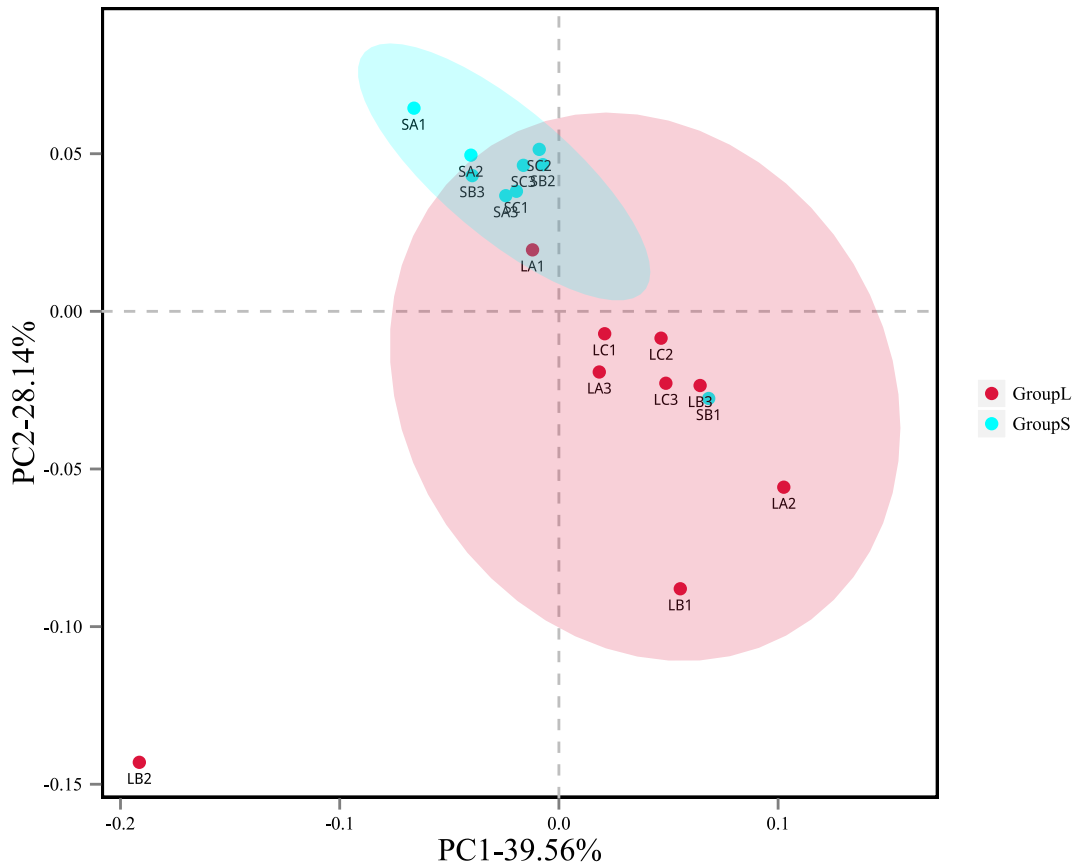

Supplement: Supplementary Figure 2 — PCoA analysis of LA and SA systems at KO level (with individual label). [file Image_2.pdf]

# PCoA - PC1 vs PC2

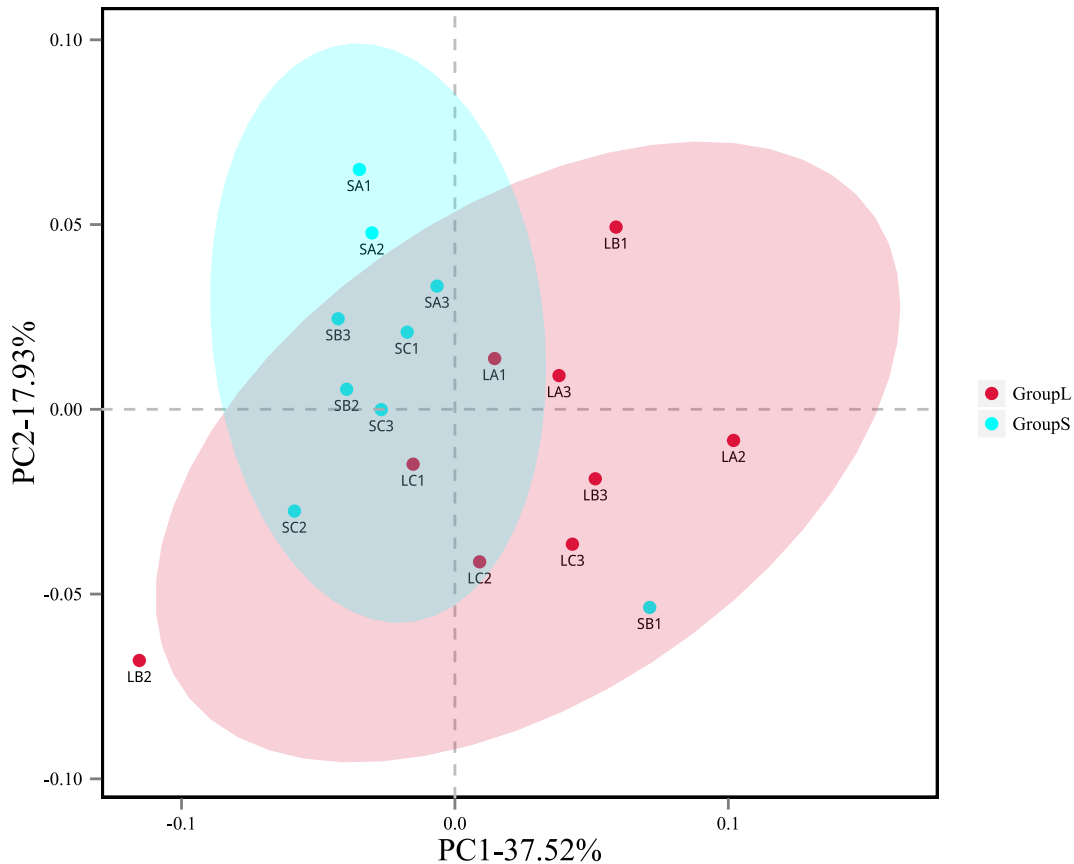

Supplement: Supplementary Figure 3 — PCoA analysis of LA and SA microorganism in CAZy-family level (with individual label). [file Image_3.pdf]
