# Supplementary material for: Metagenomic Analysis Revealed Differences in Composition and Function Between Liquid-Associated and Solid-Associated Microorganisms of Sheep Rumen
Source: Front Microbiol. 2022 May 27;13:851567. doi: 10.3389/fmicb.2022.851567 (PMC9197192; doi:10.3389/fmicb.2022.851567)
Supplement: Supplementary Figure 4 — Diagram of carbohydrate enzyme distribution in all samples (n = 18). [file Image_4.pdf]

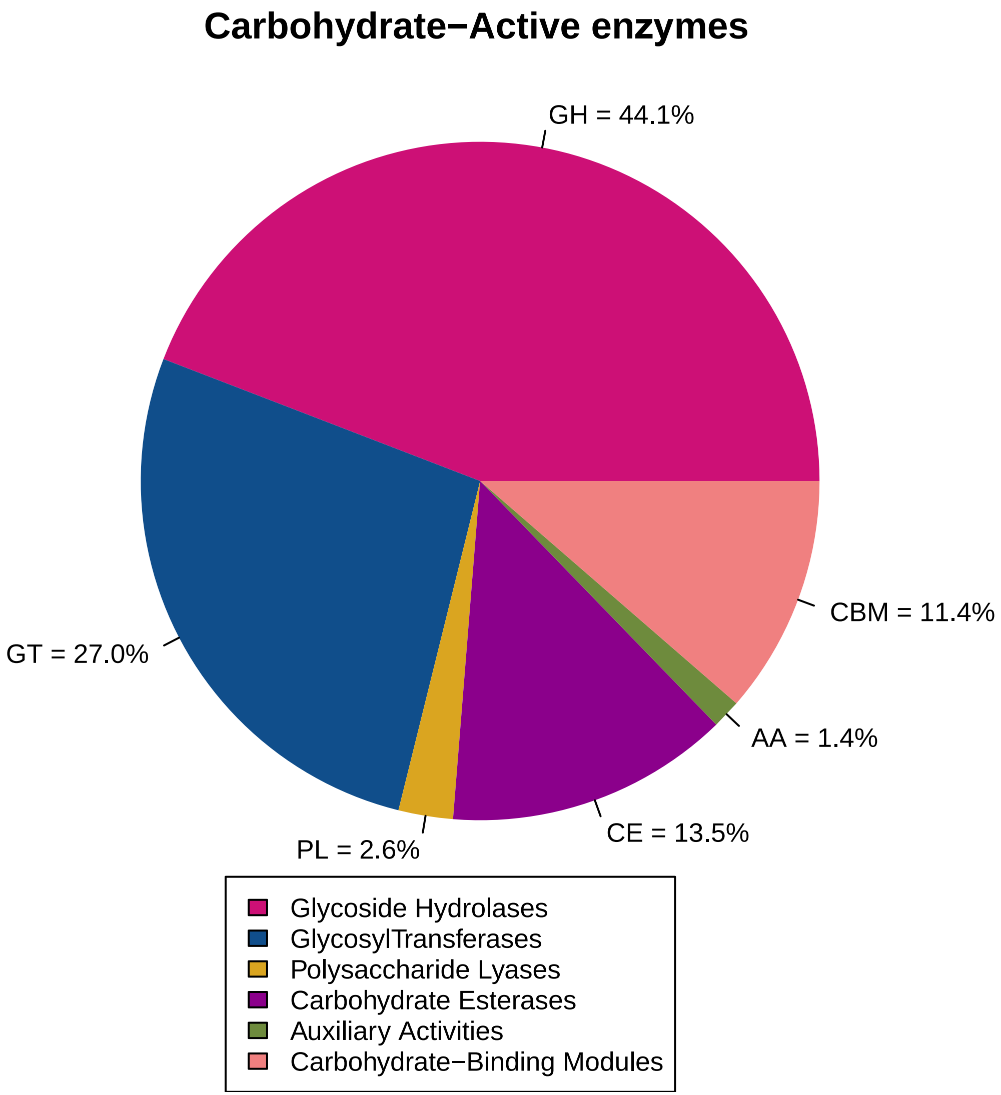

Supplementary Figure 4. Diagram of carbohydrate enzyme distribution in all samples. (n=18)
