# Supplementary material for: Metagenomic Analysis Revealed Differences in Composition and Function Between Liquid-Associated and Solid-Associated Microorganisms of Sheep Rumen
Source: Front Microbiol. 2022 May 27;13:851567. doi: 10.3389/fmicb.2022.851567 (PMC9197192; doi:10.3389/fmicb.2022.851567)
Supplement: Supplementary Figure 5 — Percent contributions of CAZymes from the microorganisms at phylum level in LA and SA groups. GH stands for glycoside hydrolase, GT for glycosyltransferase, and PL for polysaccharide lyase. CE for carbohydrate esterases, CBM for carbohydrate-binding module. [file Image_5.pdf]

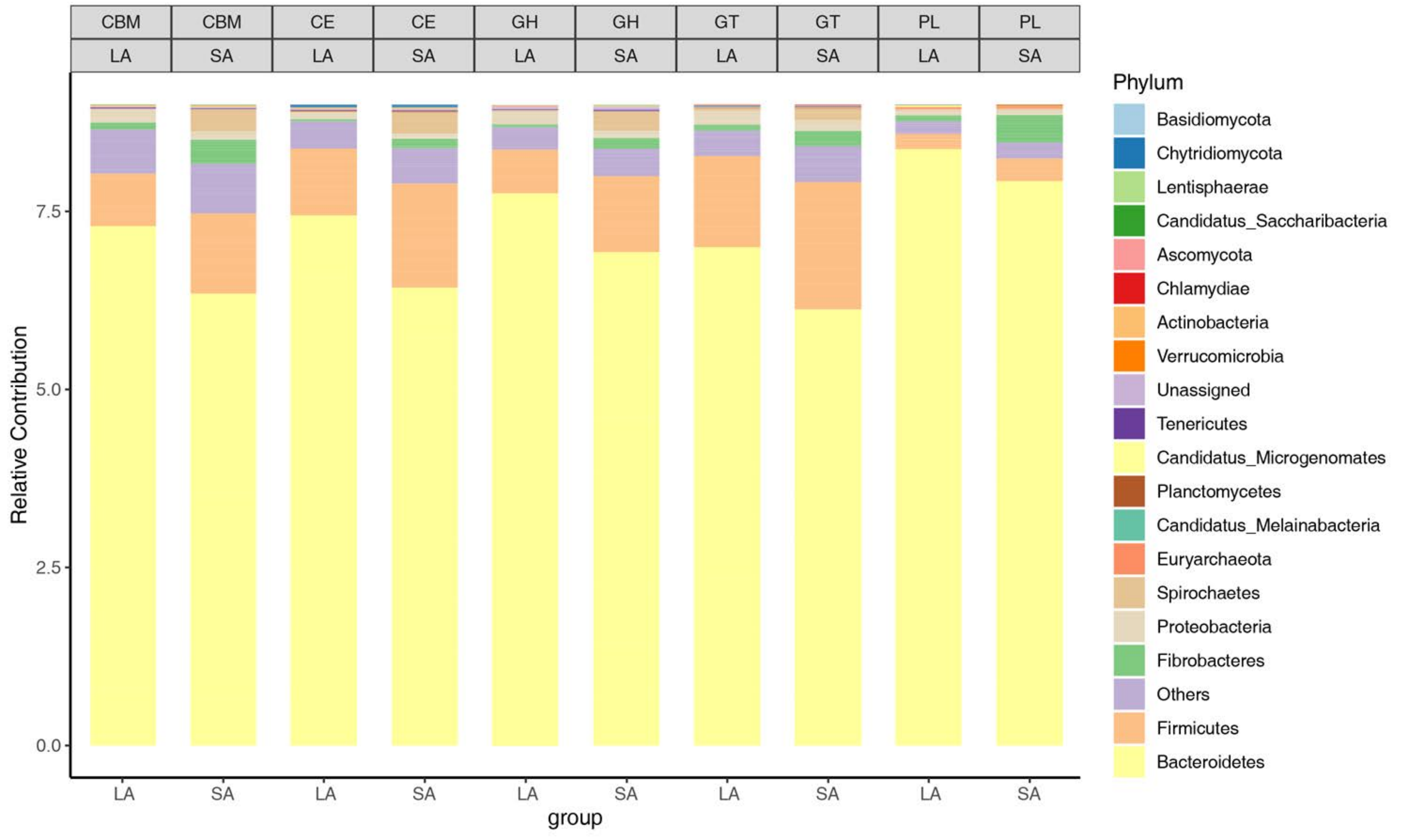
CBM
CBM
CE
CE
GH
GH
GT
GT
PL
PL
LA
SA
LA
SA
LA
SA
LA
SA
LA
SA
Relative Contribution
7.5
5.0
2.5
0.0
group
Phylum
Basidiomycota
Chytridiomycota
Lentisphaerae
Candidatus_Saccharibacteria
Ascomycota
Chlamydiae
Actinobacteria
Verrucomicrobia
Unassigned
Tenericutes
Candidatus_Microgenomates
Planctomycetes
Candidatus_Melainabacteria
Euryarchaeota
Spirochaetes
Proteobacteria
Fibrobacteres
Others
Firmicutes
Bacteroidetes
