# Supplementary material for: Metagenomic Analysis Revealed Differences in Composition and Function Between Liquid-Associated and Solid-Associated Microorganisms of Sheep Rumen
Source: Front Microbiol. 2022 May 27;13:851567. doi: 10.3389/fmicb.2022.851567 (PMC9197192; doi:10.3389/fmicb.2022.851567)
Supplement: Supplementary Figure 7 — Venn diagram of the microbial composition of SA and LA systems at genus level. [file Image_7.pdf]

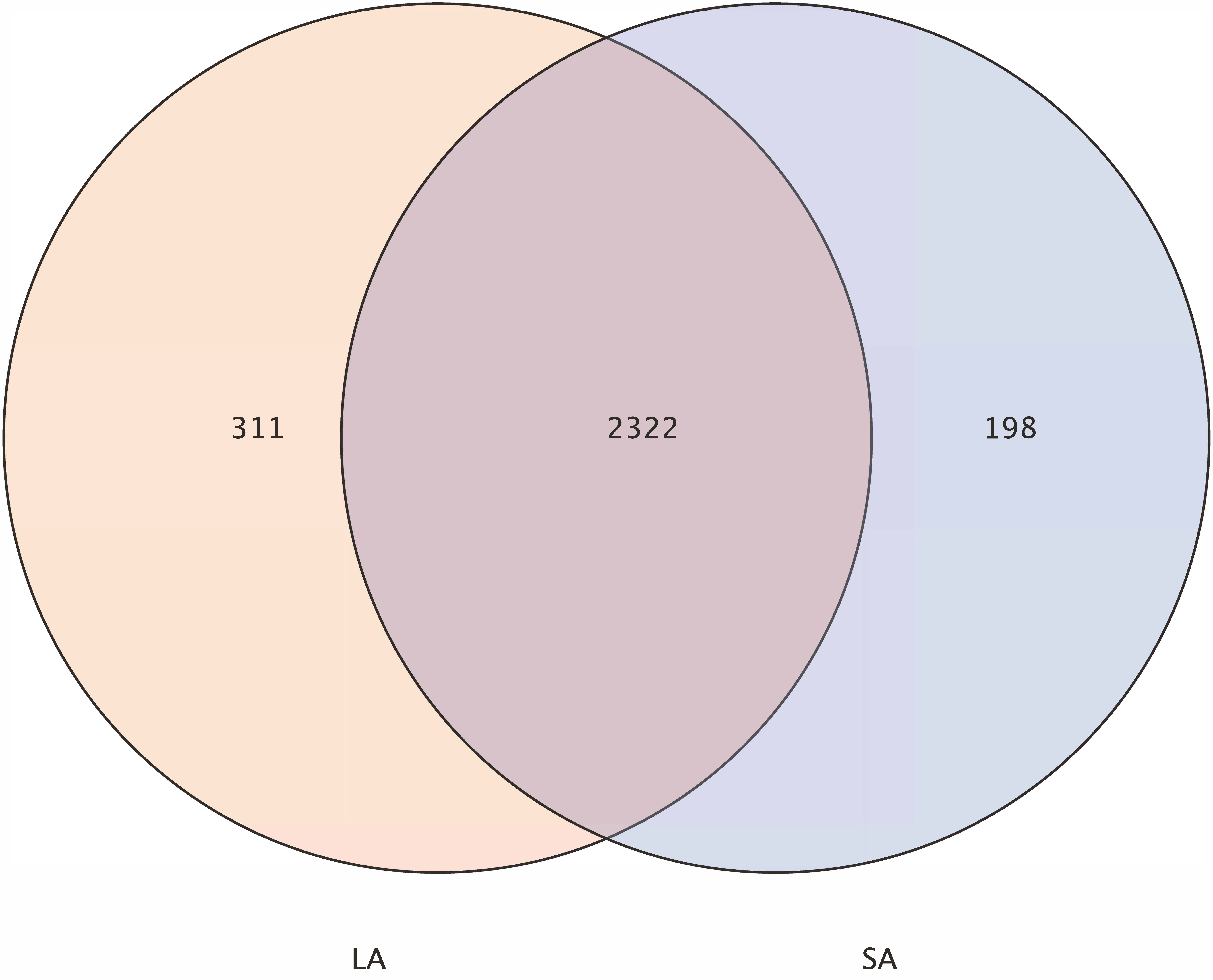

Supplementary Figure 7. Venn diagram of microbial composition of SA and LA systems at genus level.
